# Evaluation of Transarterial Chemoembolization Protocol with Drug-Eluting Beads in Combination with Lipiodol for Hepatocellular Carcinoma: A Single-Center Controlled Study

**DOI:** 10.1155/2022/1090313

**Published:** 2022-12-14

**Authors:** Xiangjun Dong, Ying Wang, Jieya Hao, Lei Chen, Tao Sun, Weihua Zhang, Bo Sun, Licheng Zhu, Yusheng Guo, Chuansheng Zheng

**Affiliations:** ^1^Department of Radiology, Union Hospital, Tongji Medical College, Huazhong University of Science and Technology, Wuhan 430022, China; ^2^Hubei Provinve Key Laboratory of Molecular Imaging, Wuhan 430022, China; ^3^Wuhan Fourth Hospital, Puai Hospital, Tongji Medical College, Huazhong University of Science and Technology, Wuhan, China

## Abstract

**Objectives:**

To evaluate the efficacy and safety of transarterial chemoembolization (TACE) with drug-eluting beads (DEB-TACE) and lipiodol (DEB-Lipiodol TACE) in the treatment of unresectable hepatocellular carcinoma (HCC) patients.

**Materials and Methods:**

The medical records of consecutive unresectable HCC patients who underwent DEB-TACE or DEB-Lipiodol TACE from June 2016 to July 2021 were retrospectively evaluated. Therapeutic response, overall survival (OS), progression-free survival (PFS), and adverse events (AEs) were compared among the groups.

**Results:**

Three hundred and twenty-seven patients were enrolled in the study, including 293 patients in the DEB-TACE group and 34 patients in the DEB-Lipiodol TACE group. The objective response rate in the DEB-Lipiodol TACE group was 17.6%, significantly higher than that in the DEB-TACE group (5.8%, *P*=0.011). Similarly, DEB-Lipiodol TACE group also had a higher disease control rate (91.2% vs 68.6%, *P*=0.006). Median OS was 13 months (95% CI: 11.0 months and 15.0 months) and 22 months (95% CI: 17.3 months and 26.7 months) in the DEB-TACE group and DEB-Lipiodol TACE group, respectively (*P*=0.041). Meanwhile, median PFS was 7 months (95% CI: 5.2 months and 8.8 months) in the DEB-TACE group and 12 months (95% CI: 7.9 months and 16.1 months) in the DEB-Lipiodol TACE group (*P*=0.174). There was no statistically significant difference in AEs incidence among the two groups (*P* > 0.05).

**Conclusions:**

DEB-Lipiodol TACE was safe, well tolerated, and had a better efficacy compared with DEB-TACE in unresectable HCC patients.

## 1. Introduction

Transarterial chemoembolization (TACE) is the current first-choice treatment in patients with intermediate-stage hepatocellular carcinoma (HCC) according to the Barcelona Clinic Liver Cancer (BCLC) staging system, especially those presenting with large or multifocal tumors with preserved liver function, deteriorated performance status, and portal vein thrombosis or extrahepatic metastases [1, 2]. TACE improves survival in HCC patients by combining targeted chemotherapy with ischemic necrosis caused by arterial embolization [3, 4]. Unfortunately, however, there is no agreement on how to proceed with TACE, with high variability in chemotherapeutics and embolization modalities, and no clear evidence of the superiority of a particular embolic material or drug [5, 6].

Nearly 40 years ago, conventional TACE (cTACE) was introduced as a treatment modality for unresectable HCC. cTACE is a combination of lipiodol and chemotherapeutic drugs injected into the tumor-supplying artery, with lipiodol acting as a contrast agent and an embolization agent. However, chemotherapeutic drugs spread rapidly from the mixture, so the visualized area does not reflect their distribution. Furthermore, the low mechanical strength of lipiodol leads to tumor arteries' recanalization because it is quickly cleared by blood scouring [7]. To overcome the drawbacks of cTACE, drug-eluting beads (DEBs) are designed to selectively deliver large amounts of chemotherapeutic drugs into the tumor over an extended period of time, minimizing the drug's blood concentration and associated systemic effects [8, 9]. However, two prospective randomized studies demonstrated that DEB-TACE did not show an advantage over cTACE in terms of radiological response and clinical outcomes [10, 11]. DEBs have poor flowability and are difficult to infiltrate into tumor peripheral arteries, leading to the reconstruction of tumor collateral circulation, which may be the main reason affecting the efficacy of DEB-TACE in the treatment of unresectable HCC [12]. Taken together, it is urgent to explore new embolization methods to address the challenges of peripheral arterial embolization and drug-controlled release.

Since lipiodol can rapidly diffuse into the peripheral tumor arteries and disrupt the blood supply of tumors by the formation of viscous oil/water emulsion with plasma [12], in this study, after DEBs embolization of the targeting arteries, we observed that lipiodol could still diffuse into the peripheral tumor arteries. However, as far as we know, no studies have reported DEBs combined with lipiodol (DEB-Lipiodol) embolization for unresectable HCC. Thus, the purpose of this study is to evaluate the efficacy and safety of DEB-Lipiodol in the treatment of unresectable HCC.

## 2. Material and Methods

### 2.1. Study Design and Patient Selection

The present retrospective, single-center study was conducted in accordance with the principles of the Declaration of Helsinki, and all procedures performed in this study were approved by the local hospital ethics committee. Written informed consent was obtained from all patients prior to treatment.

We analyzed the electronic medical records of 403 consecutive patients with unresectable HCC who received treatment with DEB-TACE or DEB-Lipiodol TACE at our center between June 2016 and July 2021. The diagnosis of HCC depended on the diagnostic criteria of the European Association for the Study of Liver and the American Association for the Study of Liver Disease [2, 13]. The TACE treatment regimen was nominated by the multidisciplinary tumor board prior to initial TACE treatment in these patients. Meanwhile, the choice of the TACE technique (DEB-TACE or DEB-Lipiodol TACE) depends entirely on the operator's experience and preference at the time of treatment.

The inclusion criteria of this study were as follows: (1) age >18 years old; (2) Child-Pugh class A or B. Exclusion criteria were as follows: (1) patients had been treated with previous surgical, locoregional, and/or systemic treatments; (2) hepatic dysfunction or renal impairment; (3) patients have other malignancies besides HCC; (4) medical records are related to hospitalization for the initial TACE treatment, 1 month of radiological and clinical follow-up, and/or clinical data deemed sufficient for statistical analysis were not available.

### 2.2. TACE Protocol

TACE was performed based on our institutional standard protocol and has been described previously [14, 15], and it was consistent with the standard chemoembolization protocol for hepatocellular carcinoma [16]. Briefly, under local anesthesia, 5-F catheter or 2.4-F microcatheter was selected to insert the tumor-supplying arteries according to the condition of the liver and vascular anatomy. For DEB-TACE, CalliSphere beads (Jiangsu Hengrui Medicine Co., Ltd., China) of different sizes (usually 100–300 *μ*m) were loaded with epirubicin in 80 mg per vial. The nonionic contrast agent was then added to the solution, and the mixture was slowly injected into the target vessels. In this study, a maximum of two vials were used per patient. If necessary, further embolization was performed with blank microspheres until the flow was near stasis. For DEB-Lipiodol TACE, like DEB-TACE, DEBs were first injected into the target vessels, and then, lipiodol (mixed with epirubicin) was slowly injected. In order to improve the effect of embolization, further reduce the damage to normal liver tissue, and protect the liver function of patients, a microcatheter under 3F is usually used to superselect the branch of the supplying artery for embolization. After embolization, reexamination angiography of the hepatic artery was performed to confirm the devascularization.

### 2.3. Follow-Up and Repeated TACE

All patients were followed-up until 31 August 2021. Laboratory tests and abdominal contrast-enhanced CT or MR were performed 6–8 weeks after the initial TACE. Follow-up CT or MR (approximately 6–8 weeks after initial TACE) was compared with preoperative imaging to determine the objective tumor radiologic regression (ORR) and disease control rate (DCR) in the liver according to modified response evaluation criteria in solid tumors [17]. ORR was defined as complete response (CR) or partial response (PR). DCR was defined as CR, PR, or stable disease (SD). TACE was repeated if patients are with intrahepatic residual viable tumor or recurrent tumor on contrast-enhanced CT or MR images and with preserved liver function. Follow-up of all patients was conducted at a 6–8-week interval after the previous TACE.

### 2.4. Definition and Evaluation of Data

Overall survival (OS) referred to the time interval between the initial TACE and the date of death or last follow-up. Progression-free survival (PFS) referred to the period between the date of initial TACE and the date of progression for patients. Adverse events (AEs) were recorded and assessed by the Common Terminology Criteria for Adverse Events Version 5.0. In addition, postembolization syndrome, such as fever, pain, nausea, and vomiting, is not considered an AE in itself, but rather an expected outcome of embolization therapy [18].

### 2.5. Statistical Analyses

All analyses were performed by using SPSS software, Version 24.0 (IBM, Armonk, New York). Categorical data were represented by numbers with percentages and calculated using the chi-squared test. Continuous variables were presented as mean ± standard deviation and calculated using Student's *t*-test. OS and PFS were plotted by using the Kaplan–Meier method. Log-rank test was used for univariate analysis, in which variables with *P* value less than 0.10 in univariate analysis were added to multivariate analysis. *P*  <  0.05 indicated a statistically significant difference. The receiver operating characteristic (ROC) curve analysis was performed to demonstrate the diagnostic significance of hypertransaminasemia for tumor response.

## 3. Results

### 3.1. Study Population and Patient Characteristics

**Figure 1:**
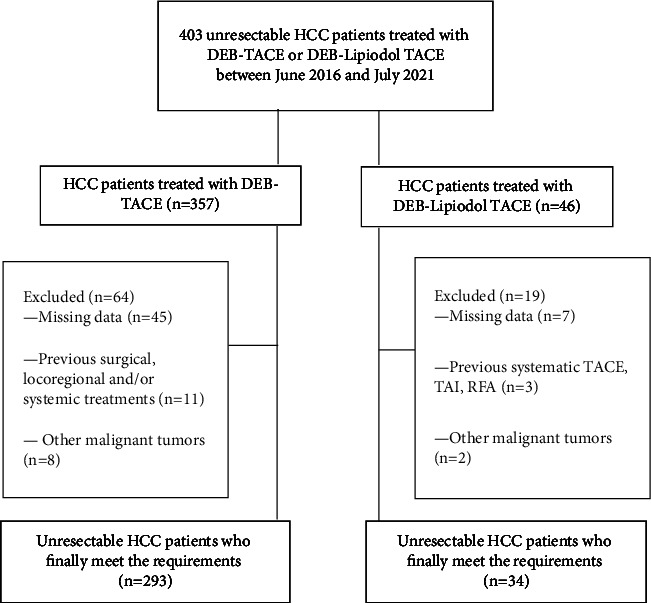
Flow chart shows the screening procedure for unresectable HCC patients treated with DEB-TACE or DEB-lipiodol TACE.

From June 2016 to July 2021, a total of 403 unresectable HCC patients received either DEB-TACE or DEB-Lipiodol TACE in our study. Of these, 76 patients were excluded because they did not meet the study requirements, as shown in Figure 1. Finally, 327 patients were enrolled in the study, 293 receiving DEB-TACE and 34 receiving DEB-Lipiodol TACE. There were no significant differences in baseline characteristics between the two groups, and detailed baseline demographics and characteristics of the 327 patients were summarized in detail in Table 1.

The median follow-up duration was 10 months (range, 1–50 months) in the DEB-TACE group and 19 months (range, 4–61 months) in the DEB-Lipiodol TACE group. In the DEB-TACE group, 187 patients (63.8%) died during the observation period, and in the DEB-Lipiodol TACE group, 19 patients (55.9%) died.

### 3.2. Safety Assessment

In the DEB-TACE group, 152 patients (51.8%) developed fever (*n* = 126), abdominal pain (*n* = 84), nausea, and vomiting (*n* = 72) within one week after TACE, and in the DEB-Lipiodol TACE group, 20 patients (58.8%) developed fever (*n* = 15), abdominal pain (*n* = 9), nausea, and vomiting (*n* = 7) within one week after TACE. In the DEB-TACE group, 26 patients (76.5%) had hypertransaminasemia, while in the DEB-Lipiodol TACE group, 210 patients (71.7%) had hypertransaminasemia. There was no significant difference between the two groups (*P*=0.555). After symptomatic treatment during hospitalization, the symptoms of all patients were relieved or significantly improved.

In the DEB-TACE group, AEs occurred after embolization in 36 patients. Eight patients developed grade 1 liver abscess, 11 patients developed grade 2 liver abscesses, and 3 patients developed grade 3 liver abscesses, which subsided after abscess drainage and antibiotic treatment. In addition, 7 patients developed grade 1 biloma, and 4 patients developed grade 2 biloma. Three patients died of liver and kidney failure 1–3 days after embolization. In the DEB-Lipiodol TACE group, AEs occurred after embolization in 5 patients, and there was no statistically significant difference in AEs incidence between the two groups (*P* > 0.05). Two patients developed grade 3 liver abscesses, which disappeared after symptomatic treatment. Three patients developed grade 2 biloma, which improved after drainage. There were no TACE-related deaths in this group.

### 3.3. Treatment Response

In the DEB-TACE group, 1 patient achieved CR, 16 patients achieved PR, and 184 patients achieved SD. Hence, the ORR and DCR were 5.8% and 68.6%, respectively. In the DEB-Lipiodol TACE group, 1 patient achieved CR, 5 patients achieved PR, and 25 patients achieved SD. Thus, the ORR and DCR were 17.6% and 91.2%, respectively. Therefore, the ORR (*P*=0.011) and DCR (*P*=0.006) of the DEB-Lipiodol TACE group were significantly better than those of the DEB-TACE group.

### 3.4. Overall Survival

**Figure 2:**
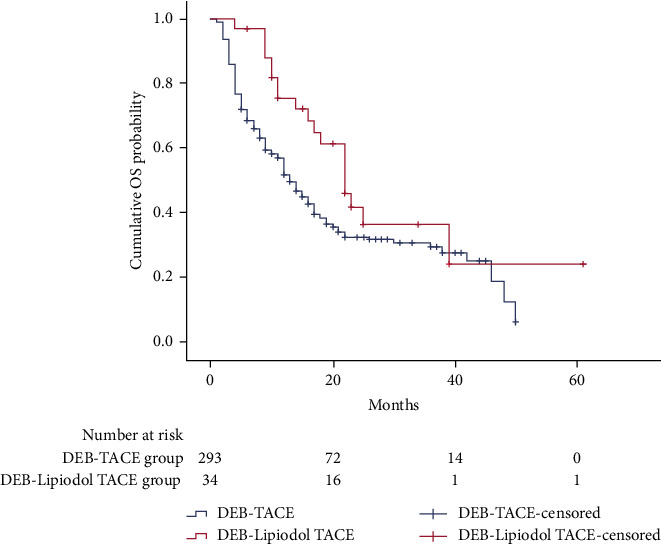
Kaplan-Meier curves of cumulative survival in HCC patients who received DEB-TACE or DEB-lipiodol TACE.

In the DEB-TACE group, the median OS was 13 months (95% CI: 11.0 months, 15.0 months). In the DEB-Lipiodol TACE group, the median OS was 22 months (95% CI: 17.3 months, 26.7 months) (Figure 2), and there was a significant statistical difference between the two groups (*P*=0.041). Univariate analysis (Table 2) indicated that the BCLC stage, aspartate aminotransferase, platelet-to-lymphocyte ratio (PLR), neutrophil-to-lymphocyte ratio (NLR), tumor size, TACE sessions, and DEB-Lipiodol TACE treatment method were associated with OS. Then, these factors were enrolled into multivariate analysis (Table 3), and we found that BCLC stage, PLR, TACE sessions, and DEB-Lipiodol TACE treatment method were independent prognostic factors affecting OS.

### 3.5. Progression-Free Survival

**Figure 3:**
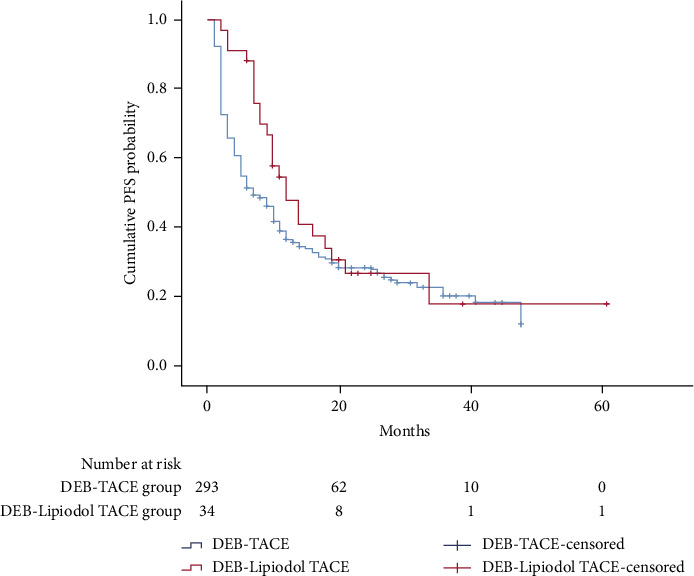
Kaplan-Meier curves of cumulative PFS in HCC patients who received DEB-TACE or DEB-lipiodol TACE.

In the DEB-TACE group, the median PFS was 7 months (95% CI: 5.2 months and 8.8 months). In the DEB-Lipiodol TACE group, the median PFS was 12 months (95% CI: 7.9 months and 16.1 months) (Figure 3), and there was no significant statistical difference between the two groups (*P*=0.174). Univariate analysis (Table 2) indicated that the BCLC stage, aspartate aminotransferase, PLR, NLR, tumor size, and TACE sessions were associated with PFS. Then, through multivariate analysis (Table 4), we found that BCLC stage and TACE sessions were independent prognostic factors affecting PFS.

### 3.6. ROC Analysis

**Figure 4:**
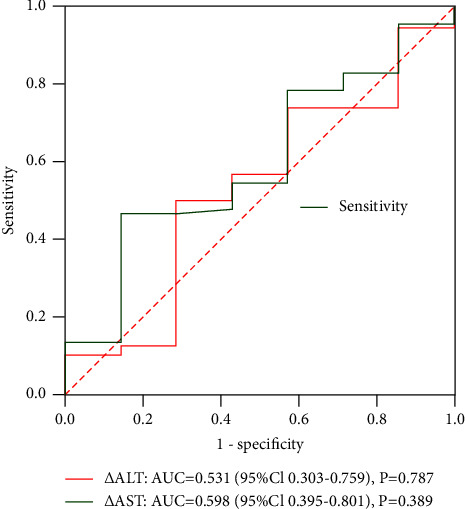
ROC curves of ΔAST and ΔALT and objective radiologic response rate.

**Figure 5:**
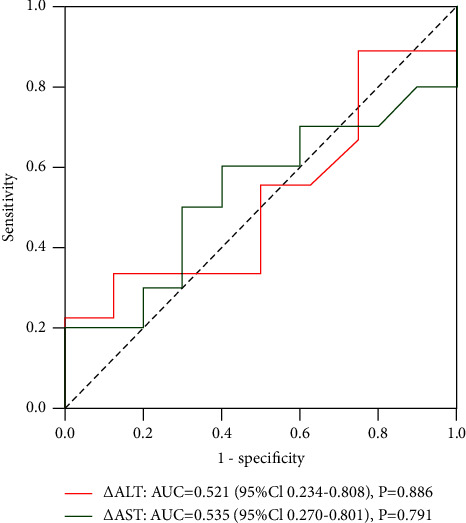
ROC curves of ΔAST and ΔALT and objective radiologic response rate in D-TACE group.

**Figure 6:**
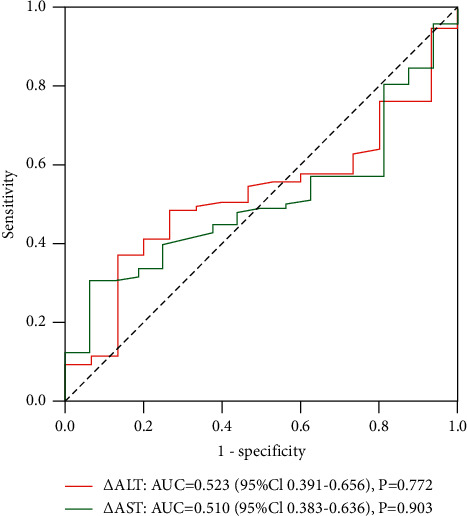
ROC curves of ΔAST and ΔALT and objective radiologic response rate in DEB-lipiodol TACE.

ROC analysis was performed on the correlation between these changes and objective remission. The results showed that no correlation was found between the objective response and post-treatment transient transaminase elevation (*P*=0.787 and *P*=0.389, respectively) (Figure 4). In addition, there was no significant correlation between postoperative hyperaminotransferemia and objective radiologic response in the D-TACE group and the DEB-Lipiodol TACE group (Figures 5 and 6).

## 4. Discussion

The present study yielded a major finding that we were able to demonstrate a positively potentiating effect of the additional use of lipiodol compared to DEB-TACE alone for unresectable HCC. The results of this study showed that compared with the DEB-TACE group, the DEB-Lipiodol TACE group had a better tumor response and a greater survival benefit.

To compensate for the deficiency of cTACE, DEBs were developed to achieve controlled drug release and permanent vascular embolization. However, DEBs have poor flowability and are difficult to infiltrate into tumor peripheral arteries, often leading to incomplete tumor embolization [12]. Insufficient embolization is known to lead to elevated levels of hypoxia in tumor tissue, which often leads to tumor angiogenesis, metastasis, and recurrence [19, 20]. Two previous prospective randomized studies also showed no significant difference in therapeutic efficacy between DEB-TACE and cTACE. Hence, in order to improve the efficacy of DEB-TACE in the treatment of unresectable HCC, DEBs and lipiodol were combined in this study to achieve complete embolization of tumor peripheral vessels. As indicated by the theoretical advantages, the ORR and DCR of the DEB-Lipiodol TACE group were significantly higher than those of the DEB-TACE group in this study, indicating that combined DEBs and lipiodol embolization can increase the rate of radiological response.

In terms of the impact of radiological response on OS, the radiological response was associated with longer OS. In the present study, DEB-Lipiodol TACE was associated with a higher rate of tumor response and increasing OS compared to DEB-TACE. This benefit in survival outcomes suggests that radiological response is one of the important determinants of long-term survival. Therefore, to put the results of this study into a clinical context, a combination of lipiodol and DEBs as an embolization regimen may be preferable to DEBs alone for patients requiring TACE as therapy of HCC. Meanwhile, the experience of the operator, local circumstances, and availability should be taken into consideration.

It has been reported that AEs were similar or decreased in DEB-TACE than cTACE, especially that the incidence of postembolization syndrome was reduced in DEB-TACE procedures [10, 11]. While in systematic reviews, AEs of DEB-TACE were similar to those of c-TACE, including postembolic syndrome [21–23], suggesting that DEB-TACE is safe. In this study, except for 3 patients in the DEB-TACE group who died of liver and kidney failure, only a few patients developed grade 1–3 AEs, and all of them improved with symptomatic treatment. Furthermore, there were no deaths in the DEB-Lipiodol TACE group and no difference in AEs incidence between the two groups, suggesting that the DEB-Lipiodol TACE group is also safe.

Recent studies have shown that the inflammation ratio of PLR may potentially serve as a quantitative biomarker for individual tumor characteristics [24, 25]. A study [26] investigated inflammatory biomarkers in patients with HCC treated with TACE and found that high baseline PLR predicted poorer tumor response and shorter PFS. Meanwhile, two other studies [27, 28] also reported that high PLR is associated with poorer OS and metastasis in HCC patients treated with TACE. In this study, multivariable analysis showed that PLR was an independent prognostic factor affecting OS. Hence, baseline PLR may be important factor affecting prognosis.

Also, note that in Table 1, we have subgrouped patients in Child-Pugh group B into B7, B8, and B9, based on a review of the benefits and harms of nontransplant therapy in patients with liver cancer and cirrhosis [29], which used Child-Pugh-Turcott (CPT) to grade liver function. TACE therapy is currently considered inappropriate for patients with hepatic decompensation, i.e., CPT B ≥8 (the presence of ascites or hyperbilirubinemia, or both), due to the high risk of severe postoperative complications. In this study, there were a total of 7 patients with CPT B ≥8 in the two groups (5 patients in the D-TACE group and 2 patients in the DEB-Lipiodol TACE group). Fortunately, no postoperative adverse events occurred in the above 7 patients after TACE treatment. Therefore, we believe that some HCC patients can receive TACE therapy under the close supervision of clinicians, even if the CPT B score ≥8. In addition, we believe that the factors affecting the therapeutic effect of TACE and the occurrence of postoperative adverse events are not only these five factors but also related to the degree of cirrhosis and tumor blood supply. As mentioned in this article, when evaluating whether CPT B HCC patients can receive TACE treatment, not only the liver function reserve but also other factors of the patient should be considered.

In addition, transient hyperaminotransferemia after cTACE has been shown to be significantly associated with objective treatment response [30]. This is inconsistent with the results of this study, and we speculate that it may be related to the sample size, the patient's HCC risk factors, and baseline liver function. Therefore, multicenter, large-sample studies are necessary to evaluate the relationship between transient hyperaminotransferemia and objective treatment response.

Our study has several limitations. First, the present study was conducted in a single institution with a small sample size, and therefore, a multicenter prospective randomized trial is needed to validate the results of this study. Second, patients and tumors may have different characteristics in different countries. Larger studies or meta-analyses in different regions may be required to demonstrate the efficacy of DEB-Lipiodol TACE in treating unresectable HCC patients. Lastly, we did not include a control group of HCC patients who received cTACE. Therefore, further comparative studies are needed to elucidate the clinical efficacy of these three embolization methods in the treatment of unresectable HCC.

## 5. Conclusion

In conclusion, this study indicated that DEB-Lipiodol TACE was able to achieve better tumor response and survival benefits in unresectable HCC patients compared with DEB-TACE. Meanwhile, DEB-Lipiodol TACE was also safe and well tolerated for HCC patients, with no severe AEs observed. Hence, on the basis of our findings, DEB-Lipiodol TACE may be a potential new embolization treatment option for unresectable HCC patients. However, further prospective randomized controlled trials are needed to validate our observations.

## Figures and Tables

**Table 1:** Baseline characteristics.

Characteristics	D-TACE group (*N* = 293)	D-TACE + lipiodolgroup (*N* = 34)	*P* value
	(No, %; mean ± SD)	(No, %; mean ± SD)	
Gender			
Male	243 (82.9%)	30 (88.2%)	0.431
Female	50 (17.1%)	4 (11.8%)	
Age (years)	56.9 ± 11.1	55.0 ± 11.8	0.353
BCLC class			
A	81 (27.6%)	12 (35.3%)	0.572
B	75 (25.6%)	9 (26.5%)	
C	137 (46.8%)	13 (38.2%)	
Hepatitis			
Hepatitis B	221 (75.4%)	27 (79.4%)	0.607
Other	72 (24.6%)	7 (20.6%)	
Child-pugh score			
A	239 (81.6%)	30 (88.2%)	0.335
B	54 (18.4%)	4 (11.8%)	
B7	49	2	
B8	4	1	
B9	1	1	
TB (*µ*mol/L)	21.0 ± 14.1	17.9 ± 7.1	0.198
Albumin (g/L)	35.4 ± 6.1	36.3 ± 5.6	0.422
PT (s)	14.1 ± 1.5	14.2 ± 1.6	0.584
AST (*µ*mol/L)	78.5 ± 100.3	88.0 ± 99.8	0.602
ALT (*µ*mol/L)	60.8 ± 99.7	63.3 ± 47.5	0.889
PLR	150.6 ± 98.1	133.9 ± 72.7	0.338
NLR	4.3 ± 6.6	3.3 ± 2.0	0.347
Tumor size (cm)	8.0 ± 4.3	9.5 ± 4.9	0.08
Tumor number			
≤3	291 (99.3%)	33 (97.1%)	0.191
>3	2 (0.7%)	1 (2.9%)	
*α*-Fetoprotein level			
>400 ng/mL	122 (41.6%)	18 (52.9%)	0.207
≤400 ng/ml	171 (58.4%)	16 (47.1%)	
Ascites			
Absent	246 (84.0%)	30 (88.2%)	0.515
Present	47 (16.0%)	4 (11.8%)	
TACE sessions	3.45 ± 2.2	3.91 ± 2.2	0.238

*Note*. D-TACE: drug-elutingbeads-transcatheter arterial chemoembolization; SD: standard deviation; BCLC: barcelona clinical liver cancer; TB: total bilirubin; PT: prothrombin time; AST: aspartate aminotransferase; ALT: alanine aminotransferase; PLR: platelet-to-lymphocyte ratio; NLR: neutrophil-to-lymphocyte ratio.

**Table 2:** Univariate analysis of prognostic factors for overall survival and progression-free survival.

Variables	OS		PFS	
	HR (95% CI)	*P* value	HR (95% CI)	*P* value
Gender				
Male	1		1	
Female	0.879 (0.604, 1.279)	0.500	0.941 (0.667, 1.328)	0.731
Age (years)	0.995 (0.983, 1.007)	0.380	0.994 (0.983, 1.005)	0.278
BCLC classs				
C	1		1	
B	0.491 (0.352, 0.686)	0.000	0.572 (0.416, 0.786)	0.001
A	0.266 (0.183, 0.386)	0.000	0.487 (0.355, 0.666)	0.000
Hepatitis				
Hepatitis B	1		1	
Other	0.926 (0.669, 1.283)	0.646	1.037 (0.773, 1.391)	0.809
Child-pugh score				
A	1			
B	1.019 (0.704, 1.475)	0.920	1.073 (0.766, 1.503)	0.683
TB (*µ*mol/L)	1.004 (0.995, 1.013)	0.353	1.005 (0.997, 1.014)	0.234
Albumin (g/L)	0.989 (0.969, 1.009)	0.261	1.001 (0.981, 1.022)	0.905
PT (s)	0.988 (0.902, 1.083)	0.801	0.969 (0.890, 1.056)	0.476
AST (*μ*mol/L)	1.002 (1.001, 1.003)	0.002	1.001 (1.000, 1.002)	0.007
ALT (*μ*mol/L)	1.000 (0.999, 1.002)	0.728	1.001 (1.000, 1.002)	0.198
PLR	1.003 (1.002, 1.004)	0.000	1.002 (1.001, 1.004)	0.001
NLR	1.032 (1.017, 1.046)	0.000	1.022 (1.007, 1.037)	0.005
Tumor size	1.067 (1.036, 1.099)	0.000	1.058 (1.030, 1.088)	0.000
Tumor number				
>3	1		1	
≤3	0.846 (0.270, 2.649)	0.774	0.932 (0.298, 2.917)	0.903
*α*-Fetoprotein level				
≥400 ng/mL	1		1	
<400 ng/ml	0.958 (0.726, 1.265)	0.764	0.950 (0.734, 1.229)	0.695
Ascites				
Present	1		1	
Absent	0.923 (0.624, 1.366)	0.688	0.990 (0.688, 1.424)	0.955
TACE sessions	0.701 (0.646, 0.761)	0.000	0.889 (0.835, 0.946)	0.000
Treatment method				
D-TACE + Liopodol	1		1	
D-TACE	1.610 (1.003, 2.583)	0.049	1.323 (0.866, 2.019)	0.195

*Note*. OS: overall survival; PFS: progression-free survival; HR: hazard ratio; CI: confidence interval; SD: standard deviation; BCLC: barcelona clinical liver cancer; TB: total bilirubin; PT: prothrombin time; AST: aspartate aminotransferase; ALT: alanine aminotransferase; PLR: platelet-to-lymphocyte ratio; NLR: neutrophil-to-lymphocyte ratio; D-TACE: drug-elutingbeads-transcatheter arterial chemoembolization.

**Table 3:** Multivariate analysis of prognostic factors for overall survival.

Variables	HR (95% CI)	*P* value
BCLC class		
C	1	
B	0.462 (0.325, 0.657)	0.000
A	0.334 (0.222, 0.503)	0.000
AST (*μ*mol/L)	1.000 (0.999, 1.001)	0.895
PLR	1.002 (1.000, 1.003)	0.015
NLR	1.007 (0.985, 1.030)	0.510
Tumor size	1.036 (1.000, 1.075)	0.053
TACE sessions	0.698 (0.642, 0.759)	0.000
Treatment method		
D-TACE + liopodol	1	
D-TACE	2.268 (1.361, 3.779)	0.002

*Note*. HR: hazard ratio; CI: confidence interval; BCLC: barcelona clinical liver cancer; AST: aspartate aminotransferase; PLR: platelet-to-lymphocyte ratio; NLR: neutrophil-to-lymphocyte ratio; D-TACE: drug-elutingbeads-transcatheter arterial chemoembolization.

**Table 4:** Multivariate analysis of prognostic factors for progression-free survival.

Variables	HR (95% CI)	*P* value
BCLC classs		
C	1	
B	0.614 (0.440, 0.856)	0.004
A	0.636 (0.449, 0.901)	0.011
AST (*μ*mol/L)	1.001 (0.999, 1.002)	0.324
PLR	1.001 (1.000, 1.003)	0.058
NLR	1.006 (0.986, 1.028)	0.556
Tumor size	1.025 (0.992, 1.058)	0.137
TACE sessions	0.901 (0.848, 0.958)	0.001

*Note.* HR: hazard ratio; CI: confidence interval; BCLC: barcelona clinical liver cancer; AST: aspartate aminotransferase; PLR: platelet-to-lymphocyte ratio; NLR: neutrophil-to-lymphocyte ratio; TACE: transcatheter arterial chemoembolization.

## Data Availability

The data analyzed during this study are available from the Electrical Medical Database of Union Hospital, Tongji Medical College, Huazhong University of Science and Technology. The data are available from the author Chuansheng Zheng upon reasonable request.

## Abbreviations

HCC: Hepatocellular carcinoma
TACE: Transarterial chemoembolization
DEB: Drug-eluting beads
BCLC: Barcelona clinic liver cancer
ORR: Objective response rate
DCR: Disease control rate
CR: Complete response
PR: Partial response
SD: Stable disease
OS: Overall survival
PFS: Progression-free survival
AEs: Adverse events.
